# NGS Technology in Monitoring the Genetic Diversity of Cytomegalovirus Strains

**DOI:** 10.17691/stm2023.15.2.04

**Published:** 2023-03-29

**Authors:** O.E. Vankova, N.F. Brusnigina, N.A. Novikova

**Affiliations:** Head Researcher, Laboratory of Metagenomics and Molecular Indication of Pathogens; Academician I.N. Blokhina Nizhny Novgorod Scientific Research Institute of Epidemiology and Microbiology of Rospotrebnadzor (Russian Federal Consumer Rights Protection and Human Health Control Service), 71 Malaya Yamskaya St., Nizhny Novgorod, 603950, Russia; Head of the Laboratory of Metagenomics and Molecular Indication of Pathogens; Academician I.N. Blokhina Nizhny Novgorod Scientific Research Institute of Epidemiology and Microbiology of Rospotrebnadzor (Russian Federal Consumer Rights Protection and Human Health Control Service), 71 Malaya Yamskaya St., Nizhny Novgorod, 603950, Russia; Professor, Head of the Laboratory of Molecular Epidemiology of Viral Infections Academician I.N. Blokhina Nizhny Novgorod Scientific Research Institute of Epidemiology and Microbiology of Rospotrebnadzor (Russian Federal Consumer Rights Protection and Human Health Control Service), 71 Malaya Yamskaya St., Nizhny Novgorod, 603950, Russia Modern molecular genetic methods, massive parallel sequencing in particular, allow for genotyping of various pathogens with the aim of their epidemiological marking and improvement of molecular epidemiological surveillance of actual infections, including cytomegalovirus infection.

**Keywords:** NGS technology, cytomegalovirus, cypomegalovirus genotyping

## Abstract

Modern molecular genetic methods, massive parallel sequencing in particular, allow for genotyping of various pathogens with the aim of their epidemiological marking and improvement of molecular epidemiological surveillance of actual infections, including cytomegalovirus infection.

**The aim of the study** is to evaluate the next-generation sequencing (NGS) technology for genotyping clinical isolates of cytomegalovirus (CMV).

**Materials and Methods:**

The object of the study were samples of biological substrates (leukocyte mass, saliva, urine) taken from patients who underwent liver and kidney transplantation. Detection of CMV DNA was carried out by a real-time PCR using commercial diagnostic AmpliSense CMV-FL test systems (Central Research Institute for Epidemiology, Moscow, Russia). DNA extraction was performed using DNA-sorb AM and DNA-sorb V kits (Central Research Institute for Epidemiology) in accordance with manufacturer’s manual. The quality of the prepared DNA library for sequencing was assessed by means of the QIAxcel Advanced System capillary gel electrophoresis system (QIAGEN, Germany). Alignment and assembly of nucleotide sequences were carried out using CLC Genomics Workbench 5.5 software (CLC bio, USA). The sequencing results were analyzed using BLAST of NCBI server.

**Results:**

CMV DNA samples were selected for genotyping. The two variable genes, *UL55*(gB) and *UL73*(gN), were used for CMV genotype determination, which was performed using NGS technology MiSeq sequencer (Illumina, USA). Based on the exploratory studies and analysis of literature sources, primers for genotyping on the *UL55*(gB) and *UL73*(gN) genes have been selected and the optimal conditions for the PCR reaction have been defined. The results of sequencing the *UL55*(gB) and *UL73*(gN) gene fragments of CMV clinical isolates from recipients of solid organs made it possible to determine the virus genotypes, among which gB2, gN4c, and gN4b were dominant. In some cases, association of two and three CMV genotypes has been revealed.

**Conclusion:**

The application of the NGS technology for genotyping cytomegalovirus strains can become one of the main methods of CMV infection molecular epidemiology, as it allows for obtaining reliable results with a significant reduction in research time.

## Introduction

At present, molecular and genetic investigation methods occupy one of the important places in the diagnosis of infectious diseases and their epidemiological surveillance. Equipping modern laboratories with automatic capillary sequenators using Sanger’s method, as well as platforms for massive parallel sequencing (next-generation sequencing, NGS) provides the possibility to perform genotyping of different causative agents for their epidemiological marking and improvement of molecular epidemiological surveillance of the actual infections including cytomegalovirus infection.

Cytomegalovirus (CMV) is one of the main causes of congenital pathology in newborns (fetal infection rate is within 6–53%, among the preterm babies — 70%), and the main cause of complication development after transplantation of hematopoietic cells, solid organs, and severe pneumonias in HIV-infected patients [1, 2].

Cytomegalovirus is a DNA virus referred to the *Herpesvirales* order, *Herpesviridae* family, *Betaherpesvirinae* subfamily, *Cytomegalovirus* genus, *Human herpesvirus 5* species. The CMV genotype is determined by the gene complex. 12 genes are known for which the following polymorphisms are characteristic: *RL5A, RL6, RL12, RL13, UL1, UL9, UL11, UL73, UL74, UL120, UL146,* and *UL139* [3-6].

Currently, various genotyping systems based on the analysis of hypervariable genes are used [3]. The most studied and recognized as potential epidemiological markers are *UL55*(gB), *UL73*(gN), *UL74*(gO), *UL144*-TNRF genes [3-5].

Genetic variability of CMV allows the virus to realize the ways of immune evasion (for example, changes of antigen epitopes), enhance the tropism for the cells of the host organism, increase the efficiency of virus replication, and alter the sensitivity to pharmaceutic preparations.

Different methods are used to identify the CMV variants contained in the biomaterials of the infected people: the analysis of DNA restriction fragment length polymorphism; Sanger fragment sequencing, real-time genotype-specific PCR, massive parallel sequencing. Preferable are the DNA analysis methods based on sequencing. They helped demonstrate that there exists a large amount of genetically diverse CMV strains in the world [7-9].

Polymorphic genes are used as an epidemiologic marker for studying virus circulation in human population. Genomic variants of CMV strains from various geographic regions may be identical, with a substantial difference in the frequency of their occurrence. Besides, there is the probability of detecting rare or new CMV variants in the regions isolated from the rest of the world [3, 7].

Considering the variability of the gene *UL55* sequence encoding gB glycogen, 7 main CMV genotypes are distinguished: gB1, gB2, gB3, gB4, gB5, gB6, gB7 [4, 10]. Substitutions of non-conservative amino acids are shown to occur in those regions which have functional or immunologic activity [4, 5]. *UL73* gene encoding the surface gN glycoprotein is also polymorphic and allows one to select 7 gN genotypes of CMV (gN1, gN2, gN3a, gN3b, gN4a, gN4b, gN4c) [3, 11]. Glycoprotein gN is the most polymorphic among the human CMV proteins [11].

Technology of NGS gives the possibility to simultaneously sequence thousands of DNA molecules increasing thereby the speed of investigation and the volume of the data obtained. Application of the NGS platform for genotyping allows one to obtain reliable results with a substantial reduction of time for their acquisition and analysis. Besides, employing NGS technology it is possible in one reaction to determine several virus strains including those which are present in minor amounts [12]. It has been known that immunocompetent patients (HIV-infected or those after organ transplantation) and newborns with congenital CMV infection are often observed to be infected by more than one CMV strain. The CMV strains persisting in the recipient’s body before organ transplantation and in the donor’s organism may reactivate in patients receiving immunosuppressive therapy after the operation. The strains in these patients may belong to one or various genotypes [8, 13].

Besides, some researchers [8, 14] have shown that patients with CMV infection, caused by association of different virus genotypes, have higher viral load requiring more time for CMV elimination.

**The aim of the study** is to evaluate the next generation sequencing technology for genotyping cytomegalovirus clinical isolates.

## Materials and Methods

Biological material for investigation (leucocyte mass, saliva, urine) was taken from patients treated at the Department of Transplantology of Privolzhsky District Medical Center of Federal Medico-Biologic Agency of Russia (Nizhny Novgorod, Russia) after liver and kidney transplantation. The clinical material was selected and transported in compliance with SanPiN 3.3686-21 “Sanitary and epidemiological requirements for the prevention of infectious diseases”.

Cytomegalovirus DNA was detected by the real-time PCR method using diagnostic AmpliSense CMV-FL test-systems (Central Research Institute for Epidemiology, Moscow, Russia). DNA was extracted with the help of DNA-sorb AM and DNA-sorb V kits (Central Research Institute for Epidemiology) according to the instructions for use. The sensitivity of the test systems, as specified in the certificate, was 1000 virions/ml.

For genotyping, 16 samples of CMV DNA were selected. CMV genotypes were determined on two variable genes, *UL55*(gB) and *UL73*(gN), using NGS MiSeq system (Illumina, USA). Based on the exploratory studies and the analysis of literature sources, 19 pairs of primers have been tested (Table 1).

**Table 1:** Characteristic of primers used for cytomegalovirus genotyping

Sequence of primers	Source
gB up1 5’ tgg aac tgg aac gtt tgg c 3’ gB lo1 5’ gca cct tga cgc tgg ttt gg 3’ gB lo1a 5’ gaa acg cgc ggc aat cgg 3’	de Albuquerque and Costa, 2003 [15]
gB up2 5’ gat ctc ctg gga tat aca gga cg 3’ gB lo2 5’ gaa tyg ctg arg gyt tga tct tg 3’ gB up3 5’ acr ttc tgg gaa gcc tcg gaa cg 3’ gB lo3 5’ gag ttc ctt gaa gac ctc tag 3’	Barbi et al., 2006 [16]
gB up4 5’ cct cat cgc tgc tgg att 3’ gB lo4 5’ tga ctc cca cca cat ctc 3’ gB up5 5’ att tgg ccc gcg acg aac at 3’ gB lo5 5’ ctc cgt act tga ggg tag tg 3’	Shepp et al., 1998 [17]
gB NF 5’ gga tct ggt gcc tgg tag tc 3’ gB NR 5’ cga ata aga tcc gta ccc tg 3’ CLZ F 5’ tgt tct ggc aag gta tca aga a 3’ CLZ R 5’ gtg aac tgc agc tgg gcg ta 3’	Grosjean et al., 2009 [18]
gB1 forward 5’ tca cca ttc ctc tcr tac gac 3’ gB1 reverse 5’ cac cat ggc tga ccg ttt gg 3’ gB2 forward 5’ ctt taa ggt acg ggt cta cca a 3’ gB2 reverse 5’ gaa ctg tag cat tgg gca aac t 3’ gB3 forward 5’ ccg gtg tga act cca cgc g 3’ gB3 reverse 5’ gat tcg ctt tca rgy gac agg 3’ gB4 forward 5’ tcg tgc aac ttc tact ca taa tg 3’ gB4 reverse 5’cgt tac gcg ttg aga gga gat 3’	de Vries et al., 2012 [19]
gB F 5’ tgg aac tgg aac gtt tgg c 3’ gB R 5’ gca cct tga cgc tgg ttt gg 3’	Chou and Dennison, 1991 [4]
gN up 5’ tgg tgt gat gga gtg gaa c 3’ gN lo 5’ tag cct ttg gtg gtg gtt gc 3’	Lisboa et al., 2012 [20]
gN 105672F 5’ cgc gac agt acc agt tga ga 3’ gN 106306R 5’ cta cac cta cgt cac cat c 3’ gN 105672F 5’ cgc gac agt acc agt tga ga 3’ gN 106179R 5’ ctt acc ccg ccg gaa cac 3’	Grosjean et al., 2009 [18]
gNF 5’ ttg ggt cgg tca aca tcg taa g 3’ gNR 5’ ggt ggt tgc agt aaa gtt ctg ga 3’	Pignatelli et al., 2003 [11]

The study design is presented in Table 2.

**Table 2:** Study design for investigating the DNA fragments of cytomegalovirus genome using NGS technology

Steps	Equipment and reagents
DNA isolation and purification	AmpliSense CMV-FL and DNA-sorb-AM kits (Central Research Institute for Epidemiology, Moscow, Russia)
Amplification of specific DNA fragments of cytomegalovirus genome	Bio-Rad DNA Engine amplificator (Bio-Rad, USA), gene-specific primers
Purification of amplification products by extraction from agarous gel	Monarch DNA Gel Extraction Kit (New England BioLabs, USA)
Determination of DNA concentration	Qubit fluorimeter (Invitrogen, Austria)
Preparation of DNA library for sequencing	Nextera XT kit (Illumina, USA)
Quality evaluation of prepared DNA library for sequencing	QIAxcel Advanced System for capillary gel electrophoresis (QIAGEN, Germany)
Sequencing of DNA library	MiSeq Reagent Kit v3 (150 cycles), MiSeq sequencer (Illumina, USA)
Alignment and assembly of nucleotide sequences (reads)	CLC Genomics Workbench 5.5 software (CLC bio, USA)
Result analysis: analysis of nucleotide sequences of *UL55*(gB) and *UL73*(gN) genes visualization of the obtained data	BLAST, NCBI server (http://www.ncbi.nlm.nih.gov/blast) Unipro UGENE, MEGA 10, ClustalX 2.0 (http://bips.ustrasbg.fr/fr/Documentation/ClustalX)

Sequencing was done using the MiSeq Reagent Kit v3 (Illumina, USA) for 150 cycles. At the first stage, the DNA library was prepared, which included DNA fragmentation with subsequent ligation of universal oligonucleotide adapters of a known sequence and indices to the obtained DNA fragments using PCR. At the second stage, each DNA fragment was amplified by PCR. With the help of the adapter sequence, a DNA fragment was hybridized with one or two primers immobilized on the hard surface and participating in PCR. The reaction mixture containing a set of enzymes and a pool of the DNA samples was introduced to the flow cell of the MiSeq system for sequencing. The obtained data array was aligned and integrated using a reference genome and *de novo*. The acquired short reads were aligned and assembled relative to reference genome by means of sequencer firmware.

The following sequences of CMV *UL55*(gB) and *UL73*(gN) genes with known genotypes taken from the GenBank database were selected as the reference ones:

full-length genomes GQ466044, HCU66425, FJ527563, BK000394, GQ121041, GQ221975, X17403, AY446894, GQ466044;

sequences of *UL55*(gB) gene: HS5GLYBM, HS5GLYBL, HS5GLYBK, X04606, HS5GLYBI, M60929;

sequences of *UL73*(gN) gene: EU686456, EU686440, AF309995, AF224677, AF390785, AF309993, AF309987, EU686430, AF390802, AF309986, AF309975, AF309974, AF310006, AF309988, AF309980, AF309975, AF309969, GU647095, GU441773, GU376726, GU376725, GU376724, GU376723, GU376721, GU376720.

## Results

Based on the analysis of the literature data, primers used by different researchers for identifying the CMV genotype on *UL55*(gB) and *UL73*(gN) genes were selected.

Primers were selected by the following criteria: matching between the primer and the analyzed gene region, purity of the PCR-generated fragment, optimal annealing temperature, the size of the fragment being obtained. Primers offered by the six works [4, 15–19] were considered for genotyping on *UL55*(gB) gene. All the authors offered a variable region located at the N-end of the gB protein as a target fragment for genotype separation. Primers suggested by de Vries et al. in 2012 [19], who recommended to use separate pairs of primers, flanking fragments 92 bp long, for each gB genotype, were excluded from the study. They used four pairs of primers for the investigation of each sample, which increased the time of the study. This approach is justified in case of determining the genotype by PCR with electrophoretic detection of the amplified fragments in the agarous gel, but is unacceptable for genotype detection by sequencing method, which we intended to employ in our study. Primers proposed by de Albuquerque and Costa in 2003 [15] flank 305 bp long variable region located nearer to the gB C-end. The remaining primer variants covered approximately the same region located at the N-end of the gB protein, the length of which varied from 256 to 522 bp. It should be noted that primer pairs for the nested PCR, suggested by Barbi et al. in 2006 [16], occupy the region previous to the region of *UL55* gene, and flank the largest 522-bp-long gene fragment. We have corrected the 5’-primer nucleotide sequence for genotyping of *UL55*(gB) gene proposed by Chou and Dennison in 1991 [4].

Variable nucleotides were replaced with degenerate ones. All primer pairs were first tested on the control AD169 CMV strain and then on clinical samples. The best results were obtained with the primers also proposed by Chou and Dennison in 1991 [4].

In order to select the optimal primers for CMV genotyping by *UL73*(gN) gene, primers proposed in works [11, 18, 20] have been analyzed. Primers proposed by Lisboa et al. in 2012 [20] were designed for the nested PCR and, after testing, appeared to be complementary to the region of *UL72* gene rather than *UL73* and therefore were excluded from the analysis. Primers suggested by Grosjean et al. in 2009 [18] cover substantially the same variable region of *UL73* gene as primers proposed by Pignatelli et al. in 2003 [11]; however, a shift is observed in the region of the primer placement and the length of the amplified fragment increases by 20 bp. The comparative analysis of the primer work efficiency on clinical samples has shown that the frequency of detecting a specific fragment using a pair of primers proposed by Pignatelli et al. [11] is substantially higher.

Thus, primers proposed by Chou and Dennison in 1991 and Pignatelli et al. in 2003 were selected for CMV genotyping on *UL55* and *UL73* genes, respectively [4, 11].

**Figure 1.**
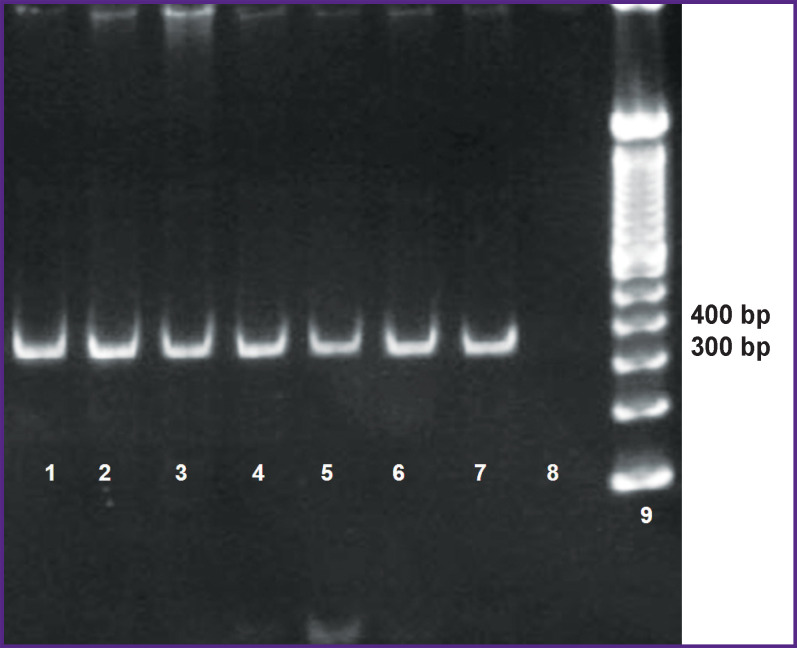
Electrophoregram of amplification products. Analysis of primer work for *UL55*(gB) gene on clinical samples The size of *UL55*(gB) gene fragment was 356 bp. Tracks 1–6 — clinical samples; track 7 — control sample; track 8 — negative control; track 9 — DNA marker

**Figure 2.**
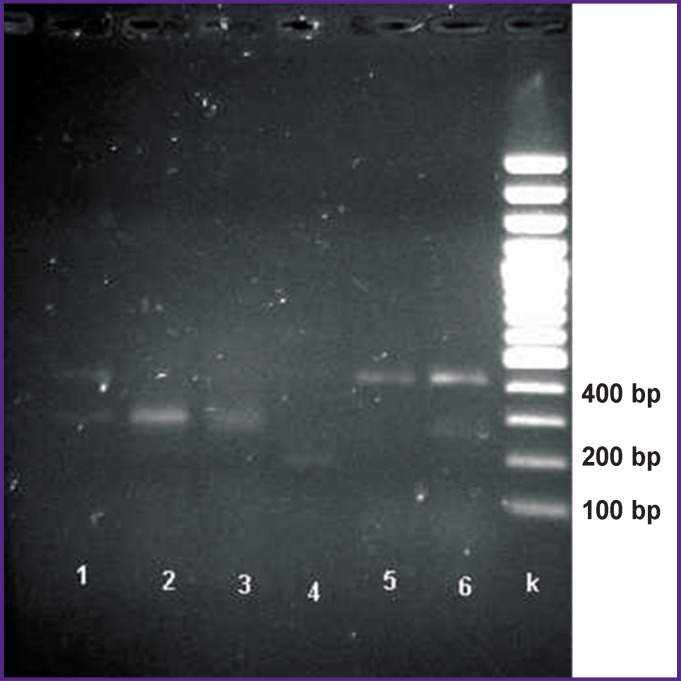
Electrophoregram of amplification products. Analysis of primer work for *UL73*(gN) gene on clinical samples The size of *UL73*(gN) gene fragment — 406 bp. Tracks 1–6 — clinical samples; k— DNA marker

In the process of work, the optimal sample volume, 10 μl, for conducting the reaction has been determined. The optimal conditions for PCR were also selected: the temperature and time of primer annealing, the number of reaction cycles. As a result, the following parameters were set: 98°C — 2 min; 98°C — 10 s; 55°C — 15 s; 72°C — 1 min; 40 cycles (Figure 1 and 2).

The analysis of *UL55* and *UL73* gene sequencing results allowed us to determine the genotype landscape of CMV circulating among the population of one region of Russia. For example, in patients who undergone transplantation of solid organs, 4 gB genotypes of CMV were identified: gB2, gB1, gB3, gB4 (in the order of occurrence frequency). Concurrent presence of two CMV genotypes, gB3 and gB4, was found for one patient.

The analysis of sequencing *UL73* fragments from the CMV isolates taken from the solid organs recipients resulted in the detection of 5 gN variants: gN4c, gN4a, gN4b, gN1, gN3b.

Simultaneous presence of several gN genotypes of CMV was identified in several patients: association of two and three genotypes was revealed in liver recipients: gN4c, gN4b and gN3b, gN4a, gN1; genotypes gN4c, gN1 and gN4c, gN4a were found in two kidney recipients.

The data obtained show that NGS technology makes it possible to perform a detailed and deep analysis of genetic variability of viral agents of infectious diseases necessary for solving both fundamental and practical tasks of epidemiology and to identify associations of various CMV genotypes in the sample of clinical material, which influences essentially the choice of etiotropic therapy.

## Discussion

Current molecular NGS technologies are the most promising and high precision methods for evaluation of genetic diversity of infectious disease agents including CMV infections.

As a result of the exploratory work, pairs of primers, reaction conditions, and design of the result analysis have been selected.

Presently, the best-studied genes, *UL55*(gB), *UL73*(gN), *UL74*(gO), *UL144*-TNRF, are used by foreign researchers as potential epidemiological markers for differentiation of clinical CMV isolates. The frequency of the CMV genotype occurrence in various geographic regions worldwide is different and is determined by the examined cohort. It has been established that gB2 genotype prevails in the HIV-infected group, while in those who undergone organ transplantation, gB1 and gN3a genotypes are encountered more often, genotypes gB1, gB2 and gN4c, gN4a genotypes dominate among children with congenital CMV infection [14, 21–24].

The selected genotyping parameters and the applied NGS technology allowed us to determine that gB2 and gN4c CMV genotypes prevailed in clinical samples collected from the recipients of solid organs. In some cases, the NGS technology made it possible to identify the CMV infection caused by the association of two and three CMV genotypes.

The obtained data show that NGS technology enables simultaneous search for the entire spectrum of CMV genotypes present in one sample and identification of both the genotype and regional structure of typical CMV population. Such investigations are necessary for examination of people in the CMV risk groups including babies in their first years of life and patients after organ transplantation. Besides, as mentioned above, the CMV infection caused by the association of several CMV genotypes may have a more severe course and require more time for virus elimination.

Investigations directed to the study of the genetic CMV diversity are needed for obtaining new knowledge on the prevalence of its different gene variants among population, improved quality of CMV infection diagnosis, effective management of risk groups.

## Conclusion

Application of NGS technology for studying genetic diversity of cytomegalovirus gives the possibility to optimize molecular monitoring of the causative agent of cytomegalovirus infection, dynamically monitor the risk groups (pregnant, newborns, children of the first year of life, and patients who undergone solid organ transplantation), predict epidemiological situation for cytomegalovirus infection, and improve the system of epidemiological surveillance of infections in general. Data on the genotypes of the circulating cytomegalovirus provide objective information about specific genotype structure of the CMV population in the region, which opens new perspectives for the development of vaccines and immunobiological preparations.
